# Pediatric ECMO Research: The Case for Collaboration

**DOI:** 10.3389/fped.2018.00240

**Published:** 2018-09-10

**Authors:** Melania M. Bembea, Aparna Hoskote, Anne-Marie Guerguerian

**Affiliations:** ^1^Department of Anesthesiology and Critical Care Medicine, Johns Hopkins University School of Medicine, Baltimore, MD, United States; ^2^Cardiorespiratory and Critical Care Division, Great Ormond Street Hospital for Children NHS Foundation Trust, London, United Kingdom; ^3^Department of Critical Care Medicine, Hospital for Sick Children and University of Toronto, Toronto, ON, Canada

**Keywords:** pediatrics, children, extracorporeal life support, extracorporeal membrane oxygenation, ECLS, ECMO, research, clinical research

## Abstract

The use of extracorporeal membrane oxygenation (ECMO) in the pediatric age has increased considerably in the last decade, as has the complexity of cases and the variety of indications outside of the neonatal age. However, no randomized controlled trials have been attempted to date to test ECMO as an intervention in non-neonatal pediatric patients with critical illness. In this review, we provide a brief overview of the history of clinical research in pediatric ECMO and discuss methodological challenges including heterogeneity of ages and diagnoses in the pediatric ECMO population, rapid advances in technology and clinical practice related to ECMO, feasibility of enrolling critically ill children on ECMO in clinical research studies, and variability in ECMO management across institutions and countries. Lastly, we discuss opportunities and existing infrastructure for future multicenter, multi-network research collaborations for pediatric ECMO studies.

## Introduction

The epidemiology of pediatric extracorporeal membrane oxygenation (ECMO) support in infants and children has undergone dramatic changes since the first ECMO case reports in the 1970's (1, 2). ECMO technology was used overwhelmingly in neonates in the first decades of its development, primarily for indications such as meconium aspiration syndrome, persistent pulmonary hypertension of the newborn, and respiratory distress syndrome. Advent of non-conventional modes of ventilation and of inhaled nitric oxide and advanced multimodal pulmonary hypertension therapies are thought to have contributed to a decline in neonatal ECMO cases in the last two decades (3, 4). A change in opposite direction occurred for ECMO utilization in older infants and children. The number of non-neonatal pediatric ECMO cases has increased as has the complexity of cases and the variety of indications (3, 4).

Overall, more than 63,000 newborns, infants and children <18 years have been reported to be supported on ECMO internationally (3). This figure represents cases reported to the Extracorporeal Life Support Organization (ELSO) registry from 1989 to present, and it is likely an underestimation of the total number of children in whom ECMO has been deployed worldwide. Modern tertiary and quaternary medical-surgical and cardiac Pediatric Intensive Care Units (PICU) rely on the ability to provide ECMO support to children with life-threatening conditions leading to cardiopulmonary failure or cardiac arrest. However, despite what has become a routine technology in the PICU, most clinical research studies conducted in pediatric ECMO have been observational in nature, with generally small sample sizes and methodological challenges stemming from the heterogeneity of patient ages, diagnoses, and equipment used, and variability in practice among ECMO providers and across institutions and countries.

In this review, we provide a brief overview of the history of clinical research in pediatric ECMO, discuss methodological challenges, and discuss opportunities for future multicenter, multi-network research collaborations for pediatric ECMO studies.

## Clinical research studies in critically ill children on ECMO

The number of PubMed indexed original studies in neonatal and pediatric ECMO has increased steadily from roughly 248 yearly in 1990 to 284 in 2000 and 377 in 2017. The overwhelming majority of these studies are observational in nature, with only a few randomized controlled trials (RCT) ever conducted.

### Studying ECMO as intervention

ECMO as intervention in children with cardiorespiratory failure or cardiac arrest has been under study for decades, primarily using registry-based case series, case control study design, or historical control cohorts (5, 6). Studies involving the random allocation of ECMO as an intervention have been rarely conducted but are clearly needed to inform the field (7). Two small RCTs (*n* = 10 and *n* = 29, respectively) of ECMO vs. standard care were conducted in the 1980s in newborns with respiratory failure (8, 9). Both trials used adaptive design and led to controversy related to the randomization schemas and the consent processes used (5, 10, 11). An additional RCT of early (oxygenation index [OI] = 25) vs. late (OI = 40) ECMO initiation was conducted at a single center (*n* = 41) with a primary outcome of cost benefit, and showed no difference in hospital charges between the two groups (12). Lastly, a RCT conducted in the U.K. in the 1990 in newborns with respiratory failure (OI ≥ 40) randomized to referral to one of five ECMO centers for consideration of ECMO vs. continued intensive conventional management at the original hospital, was stopped early due to survival advantage in the ECMO arm (13, 14). To date, no RCTs of ECMO as intervention have been conducted in children outside of the neonatal age. In fact, several landmark PICU RCTs have either excluded ECMO patients (e.g., Transfusion Strategies for Patients In Pediatric Intensive Care Units [TRIPICU] trial) (15), or considered ECMO as a study end-point (e.g., calfactant in pediatric acute lung injury trial) (16).

Controversy continues to the present time regarding the ethics of conducting a trial in which participants would be randomized to ECMO vs. continued conventional management. An excellent summary of ethical considerations for ECMO interventional trials can be found in a 2016 review by Robert Bartlett, MD (5). Some have argued that conducting a RCT of ECMO vs. continued conventional management in critically ill patients would be unethical due to the fact that those randomized to the non-ECMO arm would have higher risk of mortality. While that may hold true for refractory cardiac arrest of cardiogenic shock with impending cardiac arrest, where ECMO is used as rescue therapy, it may not hold true for certain respiratory indications for ECMO support. In adults with very severe acute respiratory distress syndrome (ARDS), the ECMO for Severe ARDS (EOLIA) trial was stopped early for a predicted lack of a significant difference in the primary outcome of mortality at 60 days between the ECMO and the continued conventional treatment arms (17). A similar trial has not been conducted in children, however recent results from a secondary analysis of the Randomized Evaluation of Sedation Titration for Respiratory Failure (RESTORE) trial suggest that a trial of ECMO vs. continued conventional management for severe pediatric ARDS may be justified (18). The authors' conclusion was based on lack of differences in patient outcomes comparing RESTORE study participants managed with ECMO vs. matched controls managed with conventional mechanical ventilation, along with the finding of wide variability in clinical practice with regards to mechanical ventilation strategies and determination of ECMO candidacy and timing for ECMO cannulation that confirmed previously published survey data (18, 19). To summarize, ECMO as intervention may become the focus of future trials in pediatrics for selected pathologies or novel indications.

### Interventional studies during the ECMO course

To date, there are no RCTs of interventions implemented during pediatric ECMO outside of the neonatal age. A single RCT has been conducted in neonatal ECMO in the U.K., investigating induced hypothermia (34°C for the first 48 to 72 h) vs. temperature of 37°C during ECMO support of newborns with acute respiratory failure (excluding neonates with congenital diaphragmatic hernia, cardiac disease, and those previously cooled for hypoxic ischemic encephalopathy) (20). The trial showed no differences in mortality, neonatal morbidity, and neurodevelopmental and behavioral outcomes at 2 years (20).

## Methodological challenges in pediatric ECMO research

ECMO is used widely in critically ill children for increasingly diverse patient populations and innovative indications (e.g., procedural ECMO) (21–23). There are now multiple reports of children with pathologies considered as contraindications in the past (e.g., pulmonary hemorrhage, malignancy, history of hematopoietic stem cell transplant, trauma, burns, etc.) being successfully supported on ECMO (24–26). It is unlikely that equipoise still exists for randomization to ECMO vs. continued conventional treatment for some ECMO indications in children (e.g., post-operative cardiogenic shock, cardiac arrest), but there is still variability in practices related to decision-making for ECMO cannulation that may be large enough to warrant ongoing evaluation for presence of equipoise for specific indications or for the timing or the means of applying the technology, as described in the previous section (e.g., pediatric ARDS) (19). That being said, methodological challenges to a RCT of ECMO vs. continued conventional treatment are numerous and potentially difficult to overcome, thus prospective data collection with data quality reviews at an international level over time may represent a better option to determine and monitor appropriate utilization of ECMO in pediatric critical care. Future efforts may be redirected to studying the optimal timing of ECMO initiation, co-interventions to be implemented during the ECMO course (e.g., mechanical ventilation strategies during ECMO, anticoagulation management and transfusion of blood products during ECMO, filtration measures, cannulation modes, nutrition, etc.), strategies for weaning and decannulation, and long-term outcomes post-ECMO.

### Heterogeneity of ages and diagnoses

Sample size calculations for ECMO-related research need to take into account the heterogeneity of ages and diagnoses of ECMO patients in contemporary era. Subgroup analyses become mandatory, as outcomes differ quite dramatically by age and diagnosis. Complicating things further, ECMO is used for rare indications that cannot be captured in conventional diagnostic categories. In an analysis of 2009-2015 ELSO registry data, the primary diagnosis of children older than 28 days cannulated onto ECMO for respiratory indications (*n* = 3,312) was asthma, bronchiolitis, pneumonia, nonpulmonary infection, drowning, inhalation, foreign body, trauma, or congenital heart disease in 57% of cases. An additional 22% of cases were classified simply as “acute respiratory failure,” and 21% of cases were classified as “other” (4). The primary diagnosis of children cannulated onto ECMO for cardiac indications during the same 7-year period (*n* = 3,850) was congenital heart disease, cardiomyopathy or myocarditis, with 26% of cases classified as “other” (4). Among neonates ≤ 28 days cannulated onto ECMO for respiratory indications (*n* = 5,839), 82% had a primary diagnosis of congenital diaphragmatic hernia, meconium aspiration syndrome, persistent pulmonary hypertension of the newborn, respiratory distress syndrome or sepsis, while 18% were classified as “other.” Lastly, among neonates ≤ 28 days cannulated for cardiac indications (*n* = 2,849), 13% had a primary diagnosis classified as “other” (4). Outcomes such as survival to ECMO decannulation and to hospital discharge differed among these four age and ECMO indication groups, as well as among subgroups within each category of ECMO indication (4).

To further determine the logistics of conducting a multicenter pediatric ECMO study with large enough representation of ages and diagnoses, an estimate of the number of potentially eligible patients requires epidemiologic data that are now available from a few sources such as the Pediatric Health Information System (PHIS) database (27), the Virtual PICU System (VPS) (28), the ELSO registry (23), or already conducted studies within pediatric research networks such as the Collaborative Pediatric Critical Care Research Network (CPCCRN) (29, 30) or the Pediatric Acute Lung Injury and Sepsis Investigators (PALISI) (31). The average number of ECMO cases per year in pediatric U.S. centers reporting data to the PHIS database between 2004 and 2011 ranged from 1 to 58 (27). Internationally, the annual hospital ECMO case volume reported to the ELSO registry between 1989 and 2013 ranged from 1 to 72 for neonatal ECMO and from 1 to 40 for pediatric ECMO (32). The median number of ECMO cases per year in neonatal and pediatric U.S. centers is 9 (3). It follows that designing adequately powered studies in pediatric ECMO within a conventional 5-year funding period could therefore only be accomplished with collaboration between a large number of centers. Limiting enrollment to specific diagnoses would bring additional challenges related to achieving an adequate sample size to answer questions of interest, although these challenges would not be insurmountable, as recently demonstrated and discussed in the case of severe pediatric ARDS (18, 33). Comparative study designs such as cluster randomized designs, adaptive designs, Bayesian designs, registry collaborative studies may provide alternative approaches, but these still require large multicenter collaborations.

### Advances in technology and clinical practice

Rapid advances in technology and clinical practice related to ECMO have the potential to confound results of ECMO studies or yield results that are no longer relevant by the end of the 7-10 years required to plan, obtain funding, and execute a multicenter RCT such as would be needed for pediatric ECMO.

A recent example is the advent of a new generation of dual lumen venovenous cannulae, which was associated with a 20% absolute increase in the proportion of venovenous cannulations and corresponding decrease in the proportion of venoarterial cannulations in children supported on ECMO for respiratory indications from 2009 to 2015 (4). Venoarterial ECMO is associated with a higher risk of complications compared to venovenous ECMO (34), so such rapid change in practice could have significantly impacted an ongoing RCT in this patient population.

New generations of extracorporeal circuits are also being developed, including surface modifications with improved biocompatibility, miniaturized circuit or circuits with self-regulated flow-demand loops (35–37). Novel therapies for multimodal pulmonary hypertension management have the potential to alter the natural history of diseases in which ECMO may be considered (38, 39). Innovative fetal therapies could similarly alter the natural history of diseases as varied as congenital diaphragmatic hernia, congenital heart disease, or congenital lung lesions (40–42). Anticoagulation management during ECMO is continually re-evaluated in terms of monitoring strategies and medications used (e.g., alternatives to unfractionated heparin) (43).

Advanced therapies and technologies are typically introduced unequally across institutions, countries, and geographic regions, based on availability of specialized equipment, personnel, monetary resources, etc. Innovations do not necessarily follow the traditional pathway of pre-clinical studies followed by knowledge translation with formal evaluation in RCTs that is only then followed by widespread implementation in humans (44). More frequently, innovations result from dramatic advances or incremental improvements in existing clinical practices that reside at the intersection of what is clearly research and what is clinical practice, the so-called “gray zone” or “zone of innovation” that is not subject to the typical human subjects research oversight (44).

### Feasibility of enrolling critically ILL children on ECMO in clinical research studies

Cardiopulmonary failure or cardiac arrest requiring ECMO is clearly a life-altering event subjecting caregivers to enormous stress. The feasibility of enrolling critically ill children requiring ECMO in a RCT has yet to be established outside of the neonatal age.

While mortality of the general PICU population has decreased considerably, both in medical-surgical PICUs (2 to 4.8%) (45, 46) as well as in pediatric cardiac ICUs (2.8%) (47), mortality of children who require ECMO support is 45%, with variations in favorable or unfavorable direction based on the primary diagnosis and indication for ECMO (4). The informed consent for ECMO studies is therefore complicated by a need for families to incorporate large amounts of information related to complex ICU and ECMO care, and consent for clinical procedures that are urgent or emergent in nature, all in the face of their child's chance of mortality having increased exponentially once the need for ECMO was identified.

Enrolling critically ill children in interventional studies requires a thoughtful approach. The pediatric critical care field has made progress and gained experience in enrolling children in large multicenter RCTs (15, 30, 31, 48, 49), some of which included ECMO patients. A recent example is the therapeutic hypothermia after in-hospital cardiac arrest trial, which required randomization within 6 h of cardiac arrest, regardless of ECMO status. More than half (55%) of children enrolled underwent ECMO after cardiac arrest and before randomization (30). Recommendations for best practices for informed consent and methods of support during the consent process have been published and should be followed for any future pediatric ECMO studies (50, 51).

### Variability in ECMO practices

Inability to adequately classify and select study participants for targeted interventions is a well described problem in critical care studies (52). In addition, some have argued that variability in multiple important ICU practices outside of the intervention under study has undermined the ability of investigators to detect differences thus leading to negative or inconclusive trials (53). ECMO care is complex and multidisciplinary in nature, with multiple stakeholders involved in the decision to cannulate as well as other aspects of care once on ECMO.

Variability in ECMO practices has been well documented. Fortunately, it lends itself to standardization at least in some aspects. While not necessarily easy to implement, modifiable aspects of ECMO care include clinical protocols for monitoring of the ECMO patient (e.g., type and frequency of blood draws and laboratory testing, neurologic monitoring modalities such as cerebral oximetry or serial cranial ultrasounds), or concomitant therapies (e.g., vasoactive infusion choices, antibiotic management, timing of procedures).

More difficult if not practically impossible to modify aspects of ECMO care include choice of ECMO equipment and of core laboratory services. ECMO equipment is expensive and, even if equipment cost would be covered by the study, the initial training and maintenance of competency for newly introduced technology for large teams of physicians, nurses, respiratory therapists, perfusionists, etc., would increase study cost significantly. Core laboratory services are also difficult to change and depend on existing equipment and resources at each participating center. As an example, anticoagulation monitoring during ECMO can range from simple, activated clotting time (ACT)-based, to complex monitoring integrating multiple tests of cellular, whole blood and plasma components of the coagulation system (54, 55). A recent study comparing two anticoagulation protocols in pediatric ECMO has brought to light potential limitations in conducting ECMO studies that entail analysis of adequacy of anticoagulation when different manufacturers and kits are used for laboratory testing of ACT, activated partial thromboplastin time (aPTT) or anti-factor Xa (56).

To summarize, variability in ECMO care could have consequences on the estimate's precision and on the impact of sample size for trials, but standardization of modifiable aspects of care could potentially be implemented in centers participating in large trials. Given complexity and variability in clinical practice in ECMO patients, study teams would need to anticipate and plan in advance for protocol deviations.

## The future of research in pediatric ECMO

Given the diversity of ECMO patients, multicenter, and, when appropriate, international, and multi-research network collaborations will be needed for prospective, adequately powered trials in pediatric ECMO. Multiple networks are stakeholders in ECMO-related clinical care, quality improvement, and research. While such networks are conceptually different in their immediate missions and obligations, there are excellent opportunities for synergy and collaboration.

Conventional RCTs, while difficult to conduct for the reasons detailed in the prior paragraphs, are still feasible in pediatric ECMO with adequate preparation and careful design. Innovative design, including registry-based studies, adaptive or stepped-wedge design, should all be considered. Several existing registries designed primarily for quality improvement purposes collect data in critically ill patients who may require ECMO, including patients with congenital heart disease (Society of Thoracic Surgeons—Congenital database; https://www.sts.org), cardiac arrest (American Heart Association Get With The Guidelines-Resuscitation [AHA GWTG-R] registry; http://www.heart.org/HEARTORG/Professional/GetWithTheGuidelines), cardiac disease requiring ICU admission (Pediatric Cardiac Critical Care Consortium; http://pc4quality.org), or children requiring ECMO support for any indications (ELSO registry; https://www.elso.org/Registry.aspx). Other registries have been developed for primary research purposes, including the Pediatric Pulmonary Hypertension Network (PPHNet) Informatics Registry (http://www.pphnet.org/registry) focused on children <18 years of age with pulmonary hypertension, or the PALISI Pediatric ECMO Outcomes Registry (PEDECOR) focused on anticoagulation and blood product management during ECMO. These registries have potential for data linkage or development of addenda for specific study purposes, once appropriate regulatory review and data use agreements are executed to ensure data confidentiality. Registry-based studies in pediatric cardiac arrest using databases such as the AHA GWTG-R have led to results that have been used successfully as evidence for clinical guideline development, in the absence of conventional clinical trials (57). A similar model could be used for ECMO care. It has been argued that RCTs in ECMO may be forgone all together in face of methodological difficulties and the fast-paced advances in technology (5, 58, 59). If RCTs are considered, pediatric volumes may render transitional random allocation comparative trials unsuitable for ECMO evaluations, with alternative study designs likely more suited to pediatric epidemiology and biology.

As the need for multicenter and multi-network collaborations for the conduct of clinical studies in ECMO patients has been recognized more by the pediatric critical care community, several initiatives for development of research infrastructure for ECMO research have emerged.

In pediatrics, the PALISI research network (www.palisi.org) and its subgroups (Pediatric ECMO subgroup [PediECMO], the Pediatric Critical Care Blood Research Network [BloodNet], the Hematopoietic Stem Cell Transplant subgroup) have developed ECMO-related working groups. The Pediatric Heart Network (PHN; http://www.pediatricheartnetwork.org) and the Congenital Diaphragmatic Hernia EURO Consortium (60) are examples of other research networks conducting studies in patient populations at high risk for requiring ECMO. The CPCCRN (https://www.cpccrn.org), a Eunice Kennedy Shriver National Institute of Child Health and Human Development-funded research network for pediatric critical care has conducted successful observational studies focused on ECMO (29, 61). Similar groups are starting to form internationally using the model of the International ECMO Network (ECMONet) research consortium (http://www.internationalecmonetwork.org) (7). The examples above are not meant to represent a comprehensive list; there are many other regional, national, and international organizations and networks that conduct quality improvement, educational, and research activities in children at risk for requiring ECMO support, or focused on ECMO only. Considerations for pediatric ECMO research infrastructure development include setting a pediatric ECMO research agenda, pursuing funding, establishing ground rules for data guardianship, data coordinating centers, and statistical support.

## Conclusions

Neonatal and pediatric ECMO is associated with risk and high cost but can be life-saving in selected populations. Clinical researchers in this area inevitably have to overcome challenges stemming from patient heterogeneity, variability in clinical practice, and ethical considerations. Given these challenges, alternatives to conventional RCTs should be considered. Engaging in adequately powered studies by harnessing existing national and international databases and multicenter and multi-network collaborations will have the potential to improve the quality of care of these critically ill children and improve survival with favorable long-term outcomes.

## Author contributions

MB, A-MG, and AH drafted the manuscript. All authors contributed to critical revisions of the manuscript.

### Conflict of interest statement

The authors declare that the research was conducted in the absence of any commercial or financial relationships that could be construed as a potential conflict of interest.

The reviewer GA declared a shared affiliation, with no collaboration, with one of the authors AG to the handling Editor
